# Modified negative-pressure wound therapy for linear blister formation prevention around foam dressings: technical note and case series

**DOI:** 10.1186/s13018-021-02759-x

**Published:** 2021-10-15

**Authors:** Congming Zhang, Qian Wang, Zhimeng Wang, Qiang Huang, Chenchen Zhang, Ning Duan, Hua Lin, Teng Ma, Kun Zhang, Hanzhong Xue, Zhong Li

**Affiliations:** Department of Orthopaedics and Traumatology, Hong-Hui Hospital, Xi’an Jiaotong University College of Medicine, 555 Youyi Road, Xi’an City, 710054 China

**Keywords:** Linear blister prevention, Open fracture, Surgical technique, Negative-pressure wound therapy

## Abstract

**Background:**

Linear blisters (LBs) often occur around dressings when negative-pressure wound therapy (NPWT) is used to cover open wounds. Tension blisters may increase the wound infection incidence rate, delay the start of operation, and prolong the duration of hospital stay. Currently, there are no established methods for the prevention of LB formation around dressings, which remains to be a major concern in clinical applications. Therefore, we developed a novel, simple, reproducible, and convenient method for preventing LB formation around NPWT dressings.

**Method:**

Fifty-three cases of Gustilo type II and III open fractures under NPWT were considered. NPWT was used on every wound after debridement. All patients were divided into a conventional group (27 cases, 33 wounds) and a novel group (26 cases, 27 wounds) based on the difference in the NPWT dressing appearance. A healthy volunteer with intact skin was also included to perform the detailed process of NPWT. LBs occurring on intact skin around the dressings were observed and recorded when the dressing was removed 3 days after the operation. The occurrence of LB formation and wound infection was considered as categorical data and compared between the two groups using a chi-square test. The duration of hospital stay was considered as numerical data and compared between the two groups using two independent t tests.

**Results:**

The percentage of occurrence of LB formation around dressings in the conventional group was 27.3%, whereas it was merely 3.7% in the novel group (*P* = 0.037). The infection incidence rate in the conventional group was 30.3%, whereas that in the novel group was 25.9%; however, no statistical difference was observed between the two groups (*P* = 0.708). The average duration of hospital stay in the conventional group was 14.39 ± 4.55 days, whereas that in the novel group was 11.04 ± 3.47 days (*P* = 0.003).

**Conclusion:**

Thus, changing the NPWT dressing appearance can prevent LB formation around dressings, providing an effective method to improve NPWT application. Modified NPWT dressings also shorten the duration of hospital stay, but do not significantly decrease the incidence of wound infection.

## Background

Open fractures can be defined as fractured bone exposed to contamination due to rupture of the skin and are often caused by severe trauma with a high degree of morbidity [1]. The use of negative-pressure wound therapy (NPWT) for wound treatment in open fractures was first described by Fleischmann et al. in 1995 [2]. They achieved a controllable negative pressure and constant drainage and consequently obtained a satisfactory surgical result. NPWT has predominantly been used to improve the management and rehabilitation of complicated open wounds, reduce the time required for a wound to dry, and eventually shorten the hospital stay duration [3–7]. NPWT is typically used to accelerate the healing process of superficial and deep wounds by stimulating the rapid formation of granulation tissue, increasing blood flow and bacterial clearance, and eliminating factors that block cell proliferation and repair [8]. A systematic review and meta-analysis published in 2018 [9] reported that a significantly lower infection rate, shorter wound coverage time, shorter wound healing time, shorter hospital stay duration, and lower amputation rate were observed in patients with open fractures treated with NPWT in comparison with those treated without NPWT.

Howell et al. [10] first described LB formation around dressings during NPWT application after total knee arthroplasty to reduce wound exudation and decrease the joint infection rate. They observed an LB formation rate of 63% in the NPWT test group, which was six times that of the control group treated with sterile gauze. The blisters had a linear appearance along the side of the NPWT dressing and ranged from 1 to 10 cm in length. Most blisters in the experimental group occurred on the intact skin between the tape and polyurethane ether foam. The authors suggested that the blistering could be attributed to the friction on the intact skin at the transition from the foam to the adhesive tape. The friction on the intact skin seems to be an inevitable factor in the use of NPWT. However, the authors could not provide a reliable method to protect the skin and decrease the formation of LBs. Furthermore, LB formation around dressings was also observed in patients with open fracture wounds at our hospital. To this end, we aimed to explore the mechanism of LB formation and propose a preventive measure from a different perspective.

There exist two main pathophysiological factors, namely mechanical and osmotic pressure factors, that result in common fracture blister formation. Intact skin is composed of three primary, distinct layers, including the epidermis, dermis, and subcutaneous tissue. As a mechanical factor, the dermo-epidermal junction of the skin is separated by large strains when bone fractures occur [11]. Post-traumatic edema increases the interstitial pressure and disturbs the normal cellular cohesion, causing the body fluid to flow toward the weakened dermo-epidermal junction. As an osmotic pressure factor, strains and swelling also increase the colloidal osmotic pressure in the epidermal gap; this causes the body fluid to move from low- to high-permeability areas [11–13]. In short, pressure imbalances on both sides of the dermis, whether caused by mechanical or osmotic pressure factors or both, can cause the body fluid to flow into the gap between the epidermis and dermis, resulting in fluid accumulation and blister formation.

**Fig. 1:**
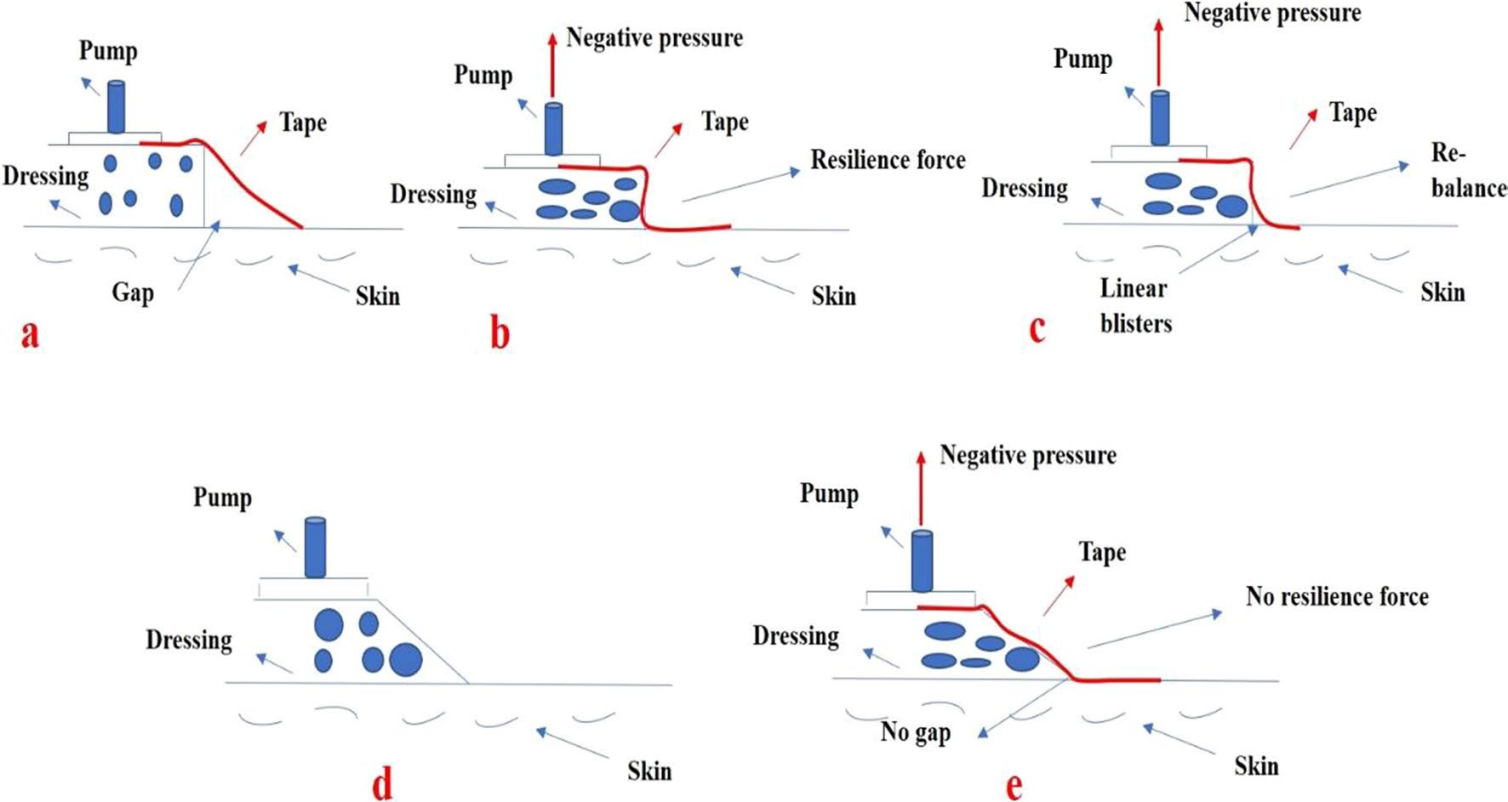
Section of conventional negative-pressure wound therapy **a** before pump operation; **b** during pump operation; and **c** after pump operation. Section of modified negative-pressure wound therapy **d** before pump operation; and **e** after pump operation

In this study, we hypothesize that the main reason for LB formation around dressings in the use of NPWT on open wounds is the gap residue between the tape, dressing, and skin (Fig. 1a). Based on our hypothesis, we proposed a simple method of changing the geometrical appearance of dressings to prevent LB formation around them (Fig. 1e, f). This paper reviews a case series clinically and presents a modified NPWT dressing technique to improve wound coverage of open fractures, thereby decreasing wound complication and hospital stay duration.

## Patients and methods

### Patients

This is a retrospective case series conducted at Xi’an Honghui Hospital. The inclusion criteria were as follows:

1. Patients with open bone fracture aged 18–65 years;
2. Patients with Gustilo–Anderson type II and III open fracture;
3. Patients with open bone fracture limited to the limbs, feet, and ankles.

The exclusion criteria were as follows:

1. Patients aged younger than 18 years or older than 65 years;
2. Patients with severe diabetes, and abnormal thyroid and renal function;
3. Patients with severe cardiopulmonary disease who cannot tolerate surgery and anesthesia.

In this study, we reviewed 53 cases of patients with open bone fracture over a period of 2 years (July 2019–July 2020) using NPWT for wound cover and drainage. There were 28 males (33 wounds) and 25 females (27 wounds), aged between 18 and 65 years. Based on the difference in the method of NPWT application, all patients were divided into two groups: a conventional group (using the traditional NPWT technique) and a novel group (using the modified NPWT dressing technique). There was no significant difference in the age, gender, Gustilo–Anderson type fracture, wound site, and average duration from injury to operation between the two groups (*p* > 0.05, Tables 1, 2).

**Table 1:** Demographics of patients with open fractures

Group	Age ($\overline{{\text{x}}}$ ± s, years)	Gender		Duration from injury to operation ($\overline{{\text{x}}}$ ± s, h)	Gustilo–Anderon type	
		Male	Female		II	III
Conventional	45.2 ± 13.2	15	12	4.9 ± 1.6	11	22
Novel	41.0 ± 10.6	14	12	5.3 ± 1.7	8	19
*t* value	1.475			0.092		
*X*^2^		1.248			0.094	
*P* value	0.087	0.264		0.395	0.759

**Table 2:** Fracture site of patients with open fractures

Group	Fracture site				
	Humerus	Ulna and radius	Femur	Tibia	Foot and ankle
Conventional	1	6	8	12	6
Novel	3	4	7	8	5
*X*^2^	0.530	< 0.001	0.022	0.303	0.001
*P* value	0.466	1.000	0.881	0.582	0.974

### Methods

Preventative antibiotics were intravenously administered to patients 30 min before surgery. All patients were anesthetized with general anesthesia and were asked to lie in the supine position. The wound was washed first with liquid soap and then with hydrogen peroxide solution and a large amount (> 3000 mL) of normal saline. Wound debridement was performed from the surface to the deeper part, removing as much inactivated and contaminated soft tissue as possible except for important blood vessels and nerves to prevent infection. To bone tissue, the free bone was completely removed, retaining as much bone membrane as possible. Then, the wound was again washed repeatedly with hydrogen peroxide, povidone-iodine, and NS.

The treatment of all fractures was determined according to the Gustilo and Anderson classification [14]. Type I injuries involve a skin laceration less than 1 cm with slight soft tissue damage, type II injuries involve a laceration greater than 1 cm with moderate soft tissue damage, and type III wounds have a laceration greater than 10 cm with severe soft tissue damage. Type II open fractures were internally fixed, and type III open fractures were temporarily fixed with an external fixator, and those involving joints were temporarily fixed with a trans-joint external fixator. All patients’ wounds of type II were closed in a one stage; however, the Gustilo type III wounds are not closed until they were completely clean through the debridement every 3 days. To prevent wound infection, the irrigation and drainage tube was implanted into the wound, and NPWT (Ilsino Wound Therapy Systems & Technologies Ltd., Taiyuan, China) was used as an assistant instrument to cover all wounds, seal the main film, connect the vacuum pump, and start suction. The contact layer over the skin surface is a non-adherent material, which is recommended to avoid skin maceration and blister formation [10, 15]. The NPWT was changed every 3 days. The standard negative pressure was 125 mmHg. All the procedures of operation and NPWT were performed by a senior surgeon.

Enrolled patients were briefed about the experiments and provided written consent before all the surgical procedures were performed. The wounds of the patients in the conventional group were covered using the traditional NPWT method. In the modified NPWT method, the dressing was trimmed before application in the novel group. This study was approved by the Ethical Committee of the Honghui Hospital, Xi’an Jiaotong University.

### Procedure for novel group

The following procedure was followed for the novel group.

**Fig. 2:**
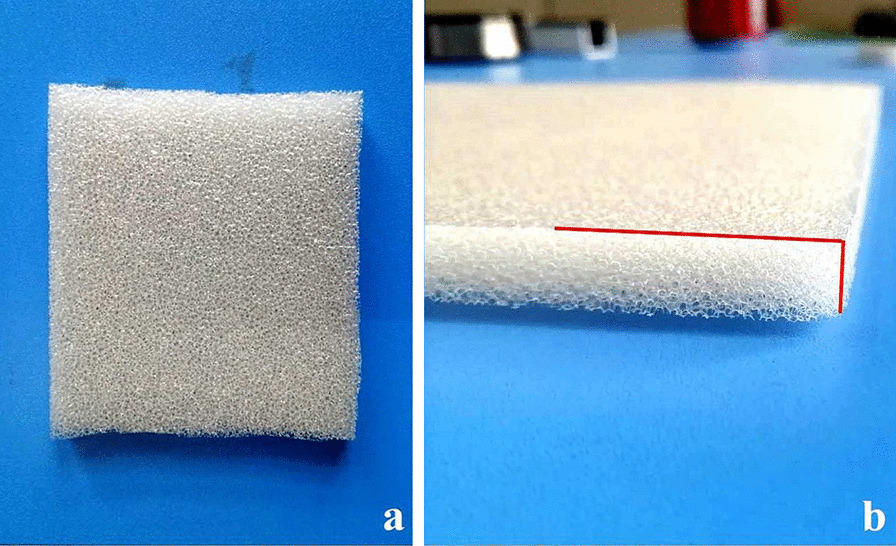
Conventional negative-pressure wound therapy with unaltered dressing edge; **a** top-down view; **b** side view. The 90° angle between the horizontal and vertical surfaces of the dressing is indicated by the red line

**Fig. 3:**
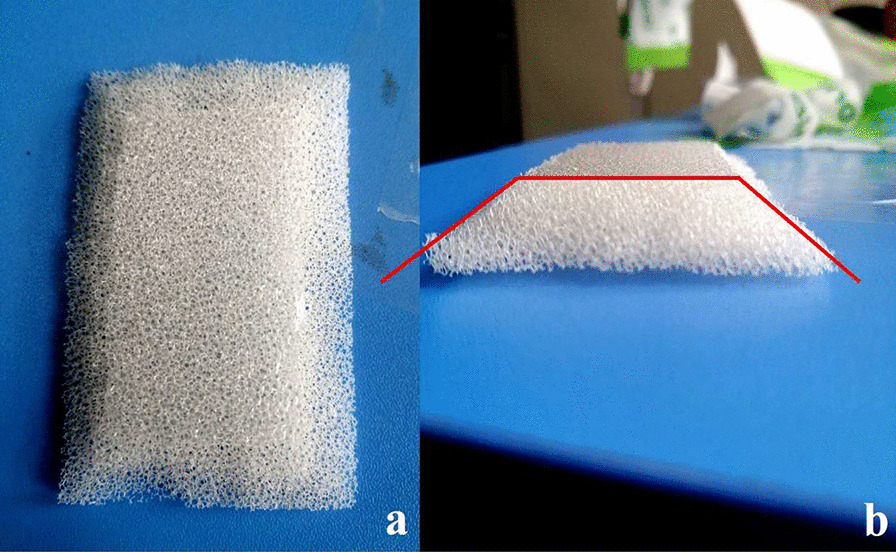
Modified dressing edge. **a** Top-down view; **b** side view. The 130° angle between the horizontal and vertical surfaces of the dressing is indicated by the red line

**Fig. 4:**
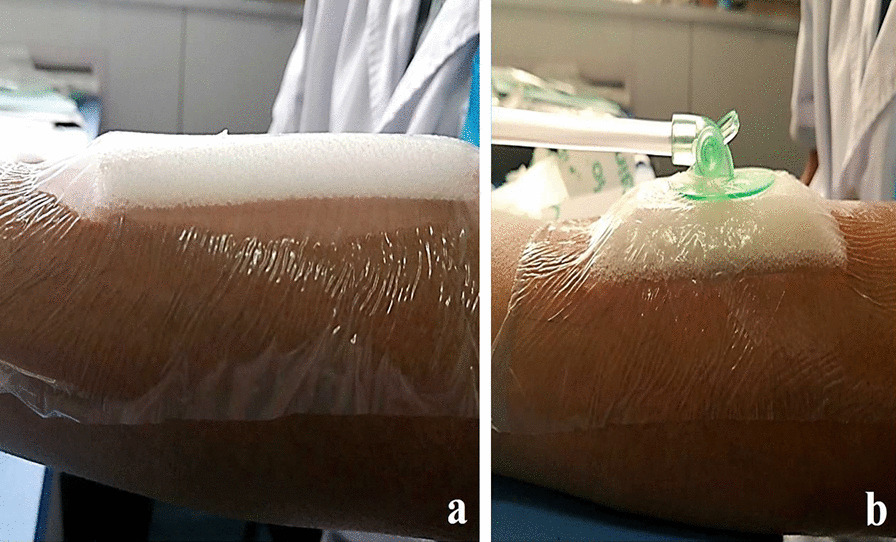
Dressing applied to the skin using tape. **a** Small amount of air remaining in the gap between the skin and dressing; **b** modified dressing showing no residual air gap between the skin, dressing, and tape

**Fig. 5:**
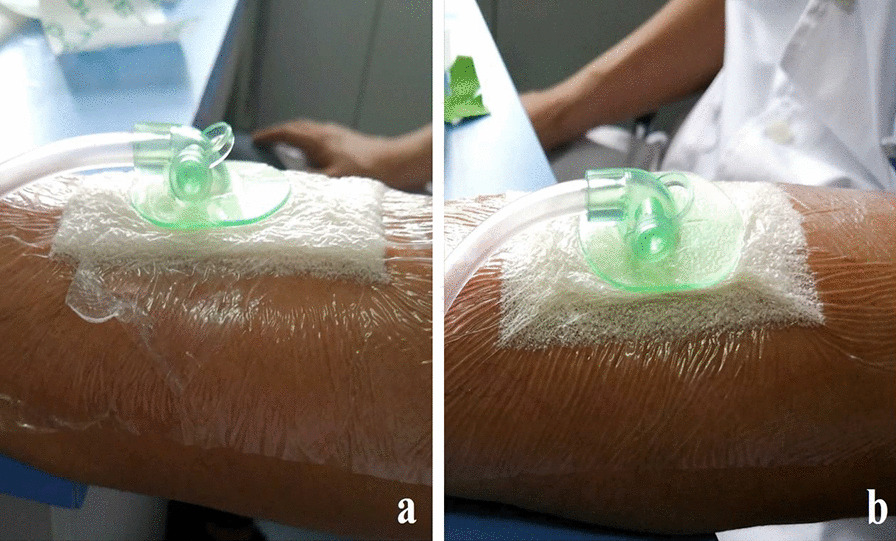
Application of negative pressure. **a** Edge of the unaltered dressing is thinned, but still retains a certain thickness; **b** thickness of the modified dressing edge is almost negligible

1. Before applying the NPWT dressing, the right angle at the edge of the foam dressing (Fig. 2a, b) was trimmed to an obtuse angle (Fig. 3a, b).
2. The tape was first applied to the skin before application to the dressing side.
3. The tape was applied evenly along the trapezoidal oblique angle (Fig. 4b).
4. Finally, the distal end of the drain was connected to an NPWT unit (Fig. 5).
5. The negative pressure was controlled within 125 mmHg once the vacuum was switched on.

### Observation indexes

Common preoperative indexes, including the wound site, Gustilo–Anderson classification, and duration from the injury to the first debridement in patients with open fractures, were compared. Additionally, the complications caused after the usage of NPWT, such as LB formation around dressings and incidence of wound infection, were compared. The incidence of LB formation around dressings was observed and recorded after 3 days of first covering the wound using NPWT. The evaluation of the incidence of wound infection was based on the method proposed by Charalambous et al. [16]. Patients were followed up with for 6 months when there was a presence of cellulitis or pus in the soft tissue area of the traumatic wound but no clinical or radiological features of osteomyelitis, requiring antibiotic treatment or surgical intervention. The above-mentioned condition is defined as superficial infection. Deep infection is defined as a clinical and radiological feature of osteomyelitis in the open fracture wound, requiring surgical bone debridement. Therefore, a positive microbiological culture is not considered to be essential for the diagnosis of superficial or deep infections. Finally, the hospital stay duration of the patients from the two groups was recorded and compared.

### Statistical methods

SPSS 22.0 software was used to process the data. All quantitative variables were expressed as mean and standard deviation. The independent samples t test was used to compare the age, duration from injury to operation, and hospital stay duration of the two groups. Bivariate data, including gender, bone fracture site, incidence of LB formation, and Gustilo–Anderson classification, were analyzed using the chi-square test. The normality of quantitative variables was tested using the Kolmogorov–Smirnov test. The equality of variances was tested using Levene’s test. *P* < 0.05 was considered to be statistically significant.

## Results

### Incidence of LB formation around dressing on wounds in both groups

A total of 10 wounds and 53 patients were identified with data on the primary outcome of LB formation. There were 9 LB formations in 33 wounds in the conventional group and 1 LB formation in 27 wounds in the novel group. Thus, the modified dressing geometry technique decreased the incidence of LB formation from 27.3% in the conventional group to 3.7% in the novel group (*P* = 0.037, Table 3).

**Table 3:** Complications and duration of hospital stay in conventional and novel groups

Group	Cases (*n*)	LB formation *n* (%)	Overall wound infection *n* (%)	Wound infection *n* (%)		Duration of hospital stay
				Superficial	Deep	
Conventional	27	9 (27.3%)	10 (30.3)	9 (27.2%)	1 (3.0%)	14.39 ± 4.55
Novel	26	1 (3.7%)	7 (25.9%)	5 (18.5)	2 (7.4%)	11.04 ± 3.47
*t* value						3.155
*X*^2^		4.364	0.140	0.636	0.032	
*P* value		0.037	0.708	0.425	0.858	0.003

### Incidence of wound infection

The overall infection incidence in the conventional group was 30.3%, which was higher than that in the novel group (25.9%). However, there was no statistical difference between the two groups (*P* = 0.708). The incidence rate of superficial infection in the conventional group was 27.2%, which was higher than that in the novel group (18.5%; *P* = 0.425). The incidence rate of deep infection in the conventional group was 3.1%, which was lower than that in the novel group (7.4%; *P* = 0.858, Table 3).

### Duration of hospital stay

The duration of hospital stay for the patients in the conventional group ranged from 5 to 23 days and that in the novel group ranged from 6 to 19 days. Thus, the average hospital stay duration of 14.39 ± 4.55 days in the conventional group was significantly higher than that in the novel group (11.04 ± 3.47 days; *P* = 0.003, Table 3).

## Case presentation

### Open fracture of distal tibia in conventional group

**Fig. 6:**
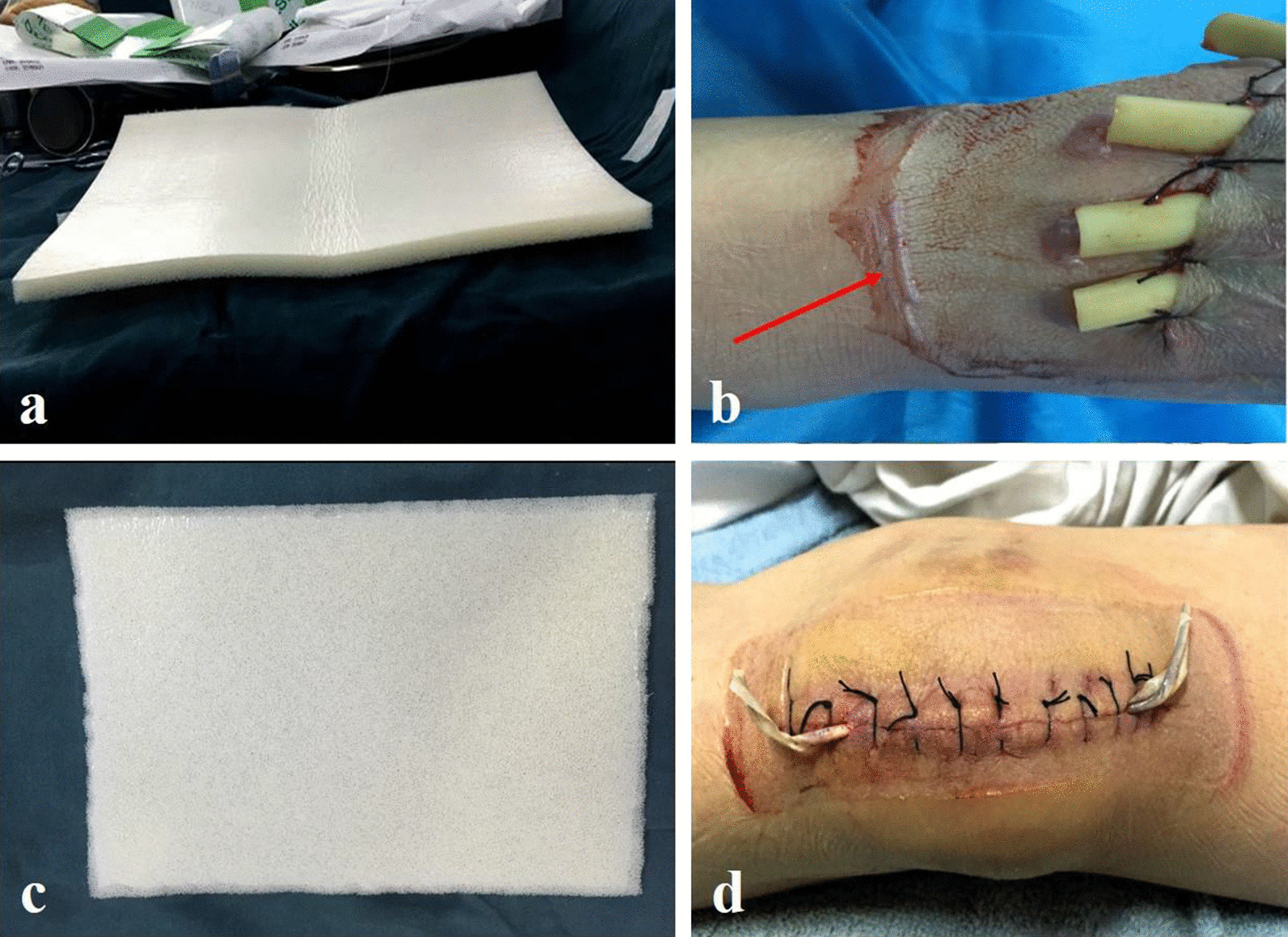
**a** Case 1: Conventional negative-pressure wound therapy was used to cover the wound. **b** Negative-pressure wound therapy dressing was removed 3 days after the operation and LB formation was observed around the dressing. **c** Modified negative-pressure wound therapy was used to clear the hematocele in the wound. **d** The negative-pressure wound therapy dressing was removed 3 days after the operation and no blisters were detected around the dressing

A 32-year-old male patient with an open foot fracture (Gustilo–Anderson classification type III) caused by a traffic accident was treated with debridement and fixation in the emergency room. The fracture was fixed using a temporary Kirschner wire, and the soft tissue was treated using traditional NPWT (Fig. 6a) without altering the edge of the dressing after debridement. The NPWT dressing was removed 3 days after the operation, and LBs were found to have formed around the edge of the dressing (Fig. 6b).

### Open fracture of patella in novel group

A 48-year-old female patient with open patellar fracture (Gustilo–Anderson classification type II) due to a fall injury was treated. After debridement, reduction, and internal fixation of the open patella fracture, NPWT was applied as an assistive treatment because of the associated heavy soft tissue injury. The edge of the NPWT dressing was trimmed into a trapezoid (Fig. 6c). When the dressing was removed 3 days after the operation, no blisters were found around the wound dressing (Fig. 6d).

## Discussion

The use of the NPWT technique is recommended after debridement and aids in covering wounds in patients with open fractures [17, 18]. However, some complications related to NPWT, including deep infection, bleeding, and graft failure, have been reported [19]. Howell et al. [10] first reported that LB formation around dressings occurred when using NPWT as an assistant instrument to suction excessive fluid from the wound. However, they could not provide a method to prevent blistering. In this study, we attributed the formation mechanism of LBs around dressings to the geometric shape of the dressing. By changing the outer shape of the dressing, we developed a simple and convenient method to reduce and prevent LB formation.

In most cases, there were no blisters beneath the dressing, while LBs were only found on the intact skin at the junction of the tape and dressing. Hence, a hypothesis was proposed in this study as follows. A triangular gap was retained between the dressing, skin, and tape (Fig. 1a) when the tape was attached to the skin and foam dressing. When the sub-atmospheric pump was operated, the air in the triangular gap was drawn away. As both sides of the film were fixed onto the skin and dressing, the tape was elongated. The resilience of the tape led to a negative-pressure formation at the junction of the dressing, skin, and adhesive tape (Fig. 1b). Furthermore, a mechanical imbalance between the triangular gap and skin was induced following the formation of the negative pressure owing to the resilience of the tape. Under the effect of this mechanical imbalance, the cellular cohesion was disturbed, and fluid accumulated in the loosened dermo-epidermal gap (Fig. 1c). When the gap filled with the negative pressure was occupied by the fluid of the LBs, the strength of the skin interface was rebalanced. The process of balancing the mechanical negative pressure to achieve re-equilibrium between the inner and outer skin led to LB formation. Therefore, only small LBs along the edge of the dressing were observed owing to the negative pressure existing only around the dressing.

Based on the theory described in this paper, a novel and simple method, which only requires the geometry of dressings to be changed, was proposed to prevent LB formation around dressings. Trimming the right angle at the edge of the dressing to an obtuse angle reduces the gap between the dressing, skin, and tape and decreases the remaining sub-atmospheric pressure (Fig. 1d). The incidence of LB formation significantly reduces owing to a lack of mechanical imbalance between the inner and outer epidermis surrounding the end of the dressing (Fig. 1e). This study provides an alternative method to prevent blister formation around dressings when using NPWT for treating open fractures.

To ensure optimal medical treatment, the negative pressure should be applied depending on the type of wound. In general, acute trauma wounds need a negative pressure of 125 mmHg [20], whereas a pressure of 50 mmHg is appropriate for chronic venous ulcers [21, 22]. Therefore, the optimal negative pressure used in our study was 125 mmHg.

The proposed technique significantly decreased the incidence of LB formation around dressings from 27.3% in the conventional group to 3.7% in the novel group. LB formation incidence in our study was lower than that observed by Howell et al. [10]. The difference in the material of the foam dressing and wound site (LB formation occurs easily at joint sites because of the lack of space caused by the soft tissue swelling) may play a key role for this discrepancy. In the conventional group, the overall infection rate was 30.3%, of which the superficial infection rate was 27.2% and the deep infection rate was 3.7%. The incidence of wound infection was similar to that in some previous studies, including soft tissue infection with an incidence rate of 0–24.4% and osteomyelitis with an incidence rate of 0–16.7% in open tibial fractures under the condition of NPWT usage [5, 23, 24]. In comparison with the incidence of wound infection in the conventional group, the overall infection rate was 25.9% (*P* = 0.708), of which the superficial infection rate was 18.5% (*P* = 0.425) and the deep infection rate was 7.4% (*P* = 0.858) (Table 3). Thus, the proposed method does not influence the incidence of wound infection in open fractures. To open fractures, the primary open wound may be a fundamental reason for wound infection. The average duration of hospital stay was 14.39 days for the conventional group compared to 11.04 days for the novel group (P = 0.003) (Table 3). As Varela et al. [12] indicated, fracture blisters can lead to delayed fracture treatment and, consequently, an increased hospital stay duration, similar to that of the patients in our study.

To the best of our knowledge, this is the first study to describe the mechanism of LB formation around dressings in patients with NPWT treatment through comparison of the clinical data of two groups. Despite its many advantages, this study has the following limitations: (1) This study is a retrospective analysis in nature and the number of patients is relatively small. (2) Although the modified NPWT technique can reduce the incidence of LB formation around dressings on wounds of open fractures, observing the effect of other types of wounds, such as burns and chronic infections, treated by this method is essential to improve results.

## Conclusion

In this study, we demonstrated that one of the most common reasons for the formation of LBs around dressings is the geometric appearance of the dressing itself. Based on this theory, we proposed a modified NPWT technique wherein the shape of the dressing edge is changed to reduce or prevent LB formation around the edge of the dressing. Furthermore, the use of the modified NPWT technology can significantly reduce the duration of hospital stay; however, it cannot significantly reduce the incidence of wound infection.

## Data Availability

The data of the present study are available from the corresponding author on request.

## Abbreviations

NPWT: Negative-pressure wound therapy
LBs: Linear blisters

## References

[1] Ali AM, Noyes D, Cogswell LK (2013). Management of open fractures of the lower limb. Br J Hosp Med (Lond).

[2] Fleischmann W, Becker U, Bischoff M, Hoekstra H (1995). Vacuum sealing: Indication, technique, and results. Eur J Orthop Surg Traumatol.

[3] Liu X, Liang J, Zhao J, Quan L (2016). Vacuum sealing drainage treatment combined with antibiotic-impregnated bone cement for treatment of soft tissue defects and infection. Med Sci Monit.

[4] Wang Z, Qu W, Liu T (2016). A two-stage protocol with vacuum sealing drainage for the treatment of type c pilon fractures. J Foot Ankle Surg.

[5] Liu DS, Sofiadellis F, Ashton M, MacGill K, Webb A (2012). Early soft tissue coverage and negative pressure wound therapy optimises patient outcomes in lower limb trauma. Injury.

[6] Schlatterer DR, Hirschfeld AG, Webb LX (2015). Negative pressure wound therapy in grade IIIB tibial fractures: fewer infections and fewer flap procedures. Clin Orthop Relat Res.

[7] DeFranzo AJ, Argenta LC, Marks MW (2001). The use of vacuum-assisted closure therapy for the treatment of lower-extremity wounds with exposed bone. Plast Reconstr Surg.

[8] Lambert KV, Hayes P, McCarthy M (2005). Vacuum assisted closure: a review of development and current applications. Eur J Vasc Endovasc Surg.

[9] Liu X, Zhang H, Cen S, Huang F (2018). Negative pressure wound therapy versus conventional wound dressings in treatment of open fractures: a systematic review and meta-analysis. Int J Surg.

[10] Howell RD, Hadley S, Strauss E, Pelham FR (2011). Blister formation with negative pressure dressings after total knee arthroplasty. Curr Orthop Pract.

[11] Giordano CP, Scott D, Koval KJ, Kummer F, Atik T, Desai P (1995). Fracture blister formation: a laboratory study. J Trauma.

[12] Varela CD, Vaughan TK, Carr JB, Slemmons BK (1993). Fracture blisters: clinical and pathological aspects. J Orthop Trauma.

[13] Bork K (1978). Physical forces in blister formation. The role of colloid osmotic pressure and of total osmolality in fluid migration into the rising blister. J Invest Dermatol.

[14] Gustilo RB, Anderson JT, JSBS classics (2002). Prevention of infection in the treatment of one thousand and twenty-five open fractures of long bones. Retrospective and prospective analyses. J Bone Joint Surg Am.

[15] Stannard JP, Gabriel A, Lehner B (2012). Use of negative pressure wound therapy over clean, closed surgical incisions. Int Wound J.

[16] Charalambous CP, Siddique I, Zenios M (2005). Early versus delayed surgical treatment of open tibial fractures: effect on the rates of infection and need of secondary surgical procedures to promote bone union. Injury.

[17] Krug E, Berg L, Lee C (2011). Evidence-based recommendations for the use of Negative Pressure Wound Therapy in traumatic wounds and reconstructive surgery: steps towards an international consensus. Injury.

[18] Ryan SP, Pugliano V (2014). Controversies in initial management of open fractures. Scand J Surg.

[19] Collinge C, Reddix R (2011). The incidence of wound complications related to negative pressure wound therapy power outage and interruption of treatment in orthopaedic trauma patients. J Orthop Trauma.

[20] Timmers MS, Le Cessie S, Banwell P, Jukema GN (2005). The effects of varying degrees of pressure delivered by negative-pressure wound therapy on skin perfusion. Ann Plast Surg.

[21] Fang R, Dorlac WC, Flaherty SF, Tuman C, Cain SM, Tracy LC (2010). Feasibility of negative pressure wound therapy during intercontinental aeromedical evacuation of combat casualties. J Trauma.

[22] Borgquist O, Ingemansson R, Malmsjö M (2011). The influence of low and high pressure levels during negative-pressure wound therapy on wound contraction and fluid evacuation. Plast Reconstr Surg.

[23] Costa ML, Achten J, Bruce J (2018). Effect of negative pressure wound therapy vs standard wound management on 12-month disability among adults with severe open fracture of the lower limb: The WOLLF Randomized Clinical Trial. JAMA.

[24] Shiroky J, Lillie E, Muaddi H, Sevigny M, Choi WJ, Karanicolas PJ (2020). The impact of negative pressure wound therapy for closed surgical incisions on surgical site infection: a systematic review and meta-analysis. Surgery.

